# Management of Onsite and Remote Communication in Oncology Hospitals: Data Protection in an Era of Rapid Technological Advances

**DOI:** 10.3390/jpm13050761

**Published:** 2023-04-28

**Authors:** Mirosława Mocydlarz-Adamcewicz, Bartosz Bajsztok, Stanislav Filip, Jiri Petera, Miroslav Mestan, Julian Malicki

**Affiliations:** 1 Department of Electroradiology, University of Medical Sciences, Fredry 10, 61-701 Poznan, Poland; 2 Greater Poland Cancer Centre, Garbary 15, 61-866 Poznan, Poland; 3 Department of Oncology and Radiotherapy, Medical Faculty and University Hospital, Charles University, Sokolska 548, 500 05 Hradec Kralove, Czech Republic; 4 1st Department of Internal Medicine, Medical Faculty and University Hospital, Charles University, Sokolska 548, 500 05 Hradec Kralove, Czech Republic

**Keywords:** personal data protection, patient privacy, oncology hospital, patient identification

## Abstract

Modern communication and information technologies are rapidly being deployed at health care institutions around the world. Although these technologies offer many benefits, ensuring data protection is a major concern, and implementation of robust data protection measures is essential. In this context, health care providers and medical care facilities must frequently make difficult decisions and compromises between the need to provide effective medical care and the need to ensure data security and patient privacy. In the present paper, we describe and discuss key issues related to data protection systems in the setting of cancer care hospitals in Europe. We provide real-life examples from two European countries—Poland and the Czech Republic—to illustrate data protection issues and the steps being taking to address these questions. More specifically, we discuss the legal framework surrounding data protection and technical aspects related to patient authentication and communication.

## 1. Introduction

At present, modern communication and information technologies are being rapidly developed and deployed around the world [1]. One of the many benefits of these technological advances is that they allow us to collect vast quantities of health-related data that can be used to offer individualised approaches to medical care [2,3,4,5]. However, patient privacy issues are a major concern. In order to protect privacy rights, the collection, storage, and analysis of personal data requires strong data protection measures such as those applied in clinical trials, in which the participants must be fully informed about the data that will be collected and how it will be used. In addition, media reports about data leaks from banks, businesses, and other entities in recent years have further raised concerns about data protection [6].

Data protection is important in all fields, but privacy is especially paramount in health care due to the sensitive nature of the data. In this regard, health care providers must do everything possible to guarantee patient privacy and data protection. The failure to adequately protect confidential data could negatively affect patients in many ways—both personally and professionally—but could also have major implications for the provider, including severe reputational damage [7]. Advances in communication tools (e.g., smartphones), including miniaturisation and increased reliability and multifunctionality, are an incentive to use these tools more widely in the hospital setting. Younger people, including both patients and staff, use smartphones every day. Data security is crucial in health care, but also in other areas such as banking, which has successfully transitioned to providing online services in a secure manner, despite the ever present risks of fraud [8,9,10,11,12]. In this context, health care providers and medical care facilities must frequently make difficult decisions and compromises between the need to provide effective medical care and the need to ensure the security and privacy of patients’ health-related data.

The purpose of the present study was to describe and evaluate key issues related to data protection systems and the safety and efficacy of health care delivery in oncology hospitals. In this study, we examined information technology (IT)-related data protection issues based on real-life examples from two European cancer centres (located in Poland and the Czech Republic) in order to illustrate these challenges. In the present article, we discuss the legal framework surrounding data protection and technical aspects related to patient authentication and communication using IT.

## 2. Materials and Methods

For the purpose of this paper, the term “data protection” is used to describe all measures in place to ensure the protection of patient privacy and the security of all patient-related data obtained during the processes associated with health care delivery.

**Figure 1:**
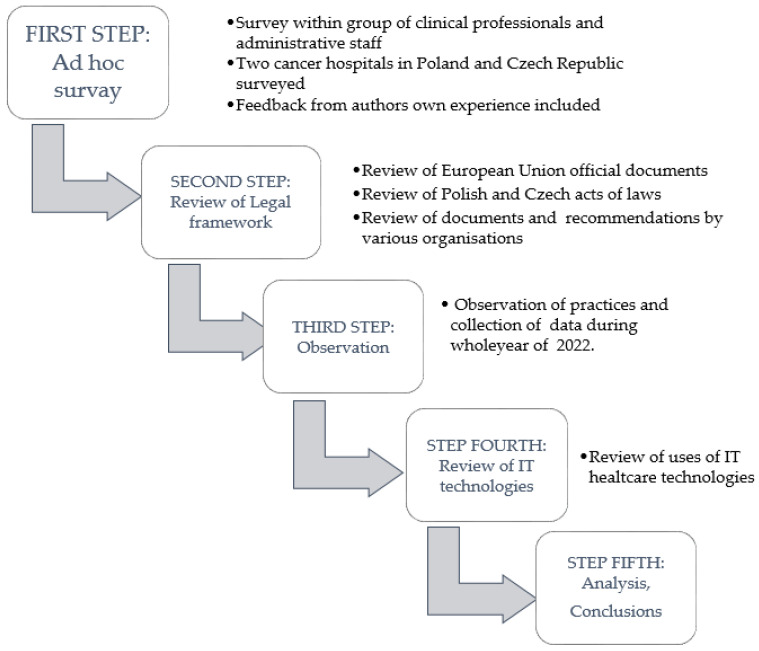
Consecutive steps in applied methodology.

The study methodology involved a five-step process (Figure 1). First, we conducted an ad hoc survey within a group of clinical professionals (nurses and physicians) and administrative staff (registration desks) at the two participating hospitals to identify the key data protection issues and challenges. In addition, the study investigators provided feedback based on their own professional experience. All of the authors of this study work in the health care field—more specifically, at cancer care hospitals—and thus have practical experience in implementing data protection in the context of clinical practice in oncology. Second, we reviewed the legal framework surrounding data protection. In this review, we considered official documents issued by the European Union (EU) and the relevant laws in Poland and the Czech Republic. We also reviewed recommendations made by professional health care organisations (e.g., medical associations, etc.) and compared the laws and binding provisions issued by the relevant regulatory bodies in Poland and the Czech Republic. The third step was an observational phase carried out during the year 2022, during which we carefully observed all practices related to the previously identified data protection issues. Fourth, we reviewed the information technologies used to facilitate healthcare delivery at the two participating hospitals in compliance with data protection regulations. Fifth, we analysed the data collected during steps 1–4, and synthesised these findings with a review and discussion of the current rules and regulations.

## 3. Results

The results shown here include key aspects related to data protection and the technologies used in Poland and the Czech Republic in accordance with current laws and legislative frameworks. Finally, the results include a discussion of how these laws impact clinical service.

**Figure 2:**
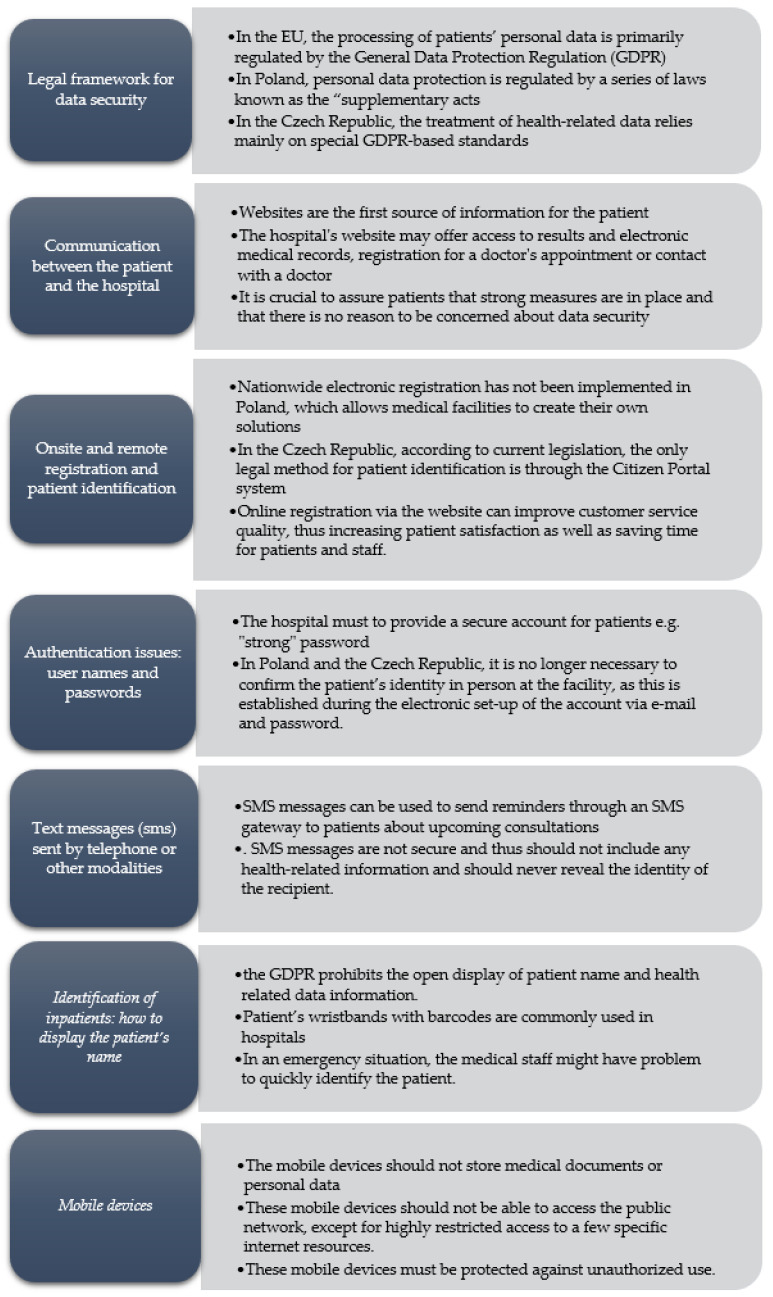
Summary of the results.

Based on the results of the staff survey, we evaluated the following six aspects: (1) communication with patients through the hospital website; (2) onsite and remote registration and patient identification; (3) authentication issues: user names and passwords; (4) text messages (sms) sent by telephone or other modalities; (5) identification of inpatients: how to display the patient’s name; and (6) issues related to the use of mobile devices. Figure 2 presents a summary of these results.

### 3.1. Legal Framework for Data Security

In the EU, the processing of patients’ personal data is primarily regulated by the General Data Protection Regulation (GDPR) [6,13]. In Poland, personal data protection is regulated by a series of laws known as the “supplementary acts” [14], particularly provisions in national law applicable to all medical facilities. The most notable of these acts are as follows: (1) the Act on the information system in health care (USIM), (2) the Act on patient rights and the patient rights ombudsman (UPP), (3) the Act on the profession of physicians and dentists, (4) the Act on the profession of nurses and midwives, and (5) the Act on medical activity.

In the Czech Republic, the treatment of health-related data relies mainly on special GDPR-based standards [15]. However, certain areas covered by existing laws, including medical records, are not impacted by the GDPR. Instead, the GDPR mostly regulates aspects such as protection of records and the requirement to shred documents and erase data after the legal retention period has expired. Nonetheless, there is a growing discussion of these questions in the Czech Republic. In fact, discussions have recently started about developing standards similar to those already in place in Poland. In this regard, it is clear that Poland is more advanced in terms of the legal regulation of the treatment of health-related information. Similar standards are just beginning to be discussed in the Czech Republic.

In Poland, medical facilities can adhere to guidelines set forth in three main documents: (1) the code of conduct for the healthcare sector [16], (2) the GDPR code of conduct for small medical facilities [17], and (3) the GDPR guidelines in healthcare (GDPR guidelines) [18]. While the first code of conduct was positively evaluated by the President of the Personal Data Protection Office in 2021, it has not yet been formally approved. The second code of conduct for small medical facilities was approved on 14 December 2022. Small medical facilities can proceed to certification and guideline implementation.

The codes of conduct provide general data processing rules for health care providers, including specific recommendations and good practices for medical facilities, which are required to appoint a “personal data controller” (ADO or controller) to oversee data protection. The ADO is also responsible for strengthening data security measures. The third document (GDPR guidelines) was created in September 2018 by the Working Group for Personal Data Protection, which was established in July 2018 by the Ministry of Digitisation. This document consists of guidelines to respond to common GDPR-related questions, including the practical application of GDPR rules in the course of routine work at medical facilities. According to Article 20 (1) of the Patients’ Rights Act, these data must be handled appropriately to guarantee privacy and data confidentiality. The GDPR guidelines clearly indicate the obligation of health care providers to adhere to all personal data protection regulations, which also obliges these entities to implement these measures without restructuring the provision of health services [19]. These provisions also stipulate that the first priority must always be the protection of human health and life; personal data protection is important, but secondary to health and safety.

The EU actively monitors data protection issues in member countries. It can apply enforcement actions to oblige countries to modify their legislation to comply with EU regulations. In this way, the EU ensures that patient privacy protections in member countries are at least equal to EU regulations.

### 3.2. Communication between the Patient and the Hospital: What Modern Hospital Websites Can Offer the Patient

Patients routinely contact medical facilities to receive information about administrative details related to health care services. Such information includes registration, the sharing of medical records with other health care providers, the rules governing the provision of health services (e.g., who is eligible for care), and information on patient rights and responsibilities. Consequently, the health care facility’s website must contain all relevant information needed by the patient. The website is often the first source of information for the patient, and the quality of the website (or lack thereof) may affect patient perceptions of the quality of the facility. A clear, well-designed website may positively influence the operation of the hospital, improving the quality of services and increasing patient trust. A good website can also be a competitive advantage [20]. For this reason, websites and associated servers should be digitally accessible to meet access criteria [21]. This means that the content presented on the website should be presented in a logical manner, with intuitive navigation to make it easy for the various stakeholders (including patients) to obtain information.

The website, apart from presenting general information about the hospital [22,23] should also provide a mechanism to allow patients to contact hospital staff. Alternatively, communication can also be carried out through a dedicated, secure portal (with robust patient identification), created specifically for this purpose. The website should offer a range of services for patients, such as online registration, access to test results and/or electronic medical records, information on the current number of patients waiting for an appointment, and the possibility to schedule an online consultation (videoconference or chat) with a physician or nurse. Adding these functions to the website, even if carried out gradually, would modernise the website to better meet patient needs over time, with the attendant social and economic benefits. However, implementing these services requires close cooperation between the hospital management team, the IT department, and specialised external providers with the knowledge and skills to create new solutions, including data protection. Nowadays, patients expect to obtain more than general information from the website, but they also expect to have access to their medical records. They also want to know how their data are processed and what security measures are in place.

Patients are increasingly aware of the need to protect their personal data. Similarly, there is also a growing awareness about the existence of data protection laws, perhaps most notably the GDPR. As a result, patients will often ask specific questions regarding how their data are processed, who has access to the data, where the data are sent, their rights regarding data processing, and what happens to the data after processing (i.e., deleted or stored) [24]. In the Czech Republic, due to regulations established by the National Cyber and Information Security Agency (NÚKIB), the provider is obligated to implement certain data protection measures. If the provider fails to properly secure the data, this could have serious legal and financial sanctions.

When patients first contact the facility, they are informed of their rights regarding data processing and access. This information is also placed on the website and/or on notice boards. Nevertheless, this does not always allay concerns about the protection of sensitive medical data. In this regard, it is crucial to assure patients that strong measures are in place and that there is no reason to be concerned about data security.

### 3.3. Onsite and Remote Registration and Patient Identification

Data processing, both for telemedicine and in-person consultations, starts as soon as the patient registers with the hospital. The data collected include all communications during the consultation and notes added to the medical records.

In most cases, the first contact between the patient and hospital starts with registration for a specific health service (e.g., appointment, examination, etc.). This process can be carried out in person at the medical facility or electronically through telephone or video. A dedicated website may be used for electronic registration (e-registration). Regardless of whether registration is completed online or in person, the process must always be completely confidential. In real-world practice, most data breaches occur during the registration process when third parties are present. One obvious example is third parties overhearing the conversation between patient and staff during the registration process, which may reveal the patient’s name and surname (and perhaps more details) [25].

In the Czech Republic, according to current legislation, the only legal method for patient identification is through the Citizen Portal system [26]. This requires the use of one of three legally accepted methods (i.e., bank identity, e-Citizen, or a special eGovernment certificate). These requirements are codified in the Act on Electronic Health Care. In Poland, all citizens have a government-issued personal identification number (PESEL). In some cases, patients are reluctant to provide certain data (e.g., name, surname, and PESEL), even though these are necessary for registration. To ensure adequate data protection during the registration process, the physical office space should be located away from the waiting room. Individual registration stations should be sufficiently separated (or located in separate rooms) to prevent the risk that a conversation could be overheard.

Electronic registration should follow the same principles indicated above. In Poland, despite the statutory obligation to facilitate automatic registration, this process is still carried out in person at many facilities. However, in many facilities, the patient can register by telephone or online (i.e., e-mail or website). The implementation of e-registration is a response to patient frustration with the in-person registration process, which can be slow due to long lines. In addition, in-person registration is more costly, both in terms of time as well as travel-related expenses.

Online registration via the website can improve customer service quality, thus increasing patient satisfaction, as well as saving time for patients and staff. At present, nationwide e-registration is not available in Poland, although it is available at many medical facilities, in some cases as a part of regional projects. The main drawback to this ad hoc process to developing an e-registration system is the lack of standardised procedures using the same (or similar) IT tools, particularly those specifically designed to ensure e-registration security. Interestingly, in the Czech Republic, individual or regional e-registration measures are prohibited by law.

All e-registration systems require the patient to set up an account with a unique username and password. An e-mail account is needed to activate the account and verify the patient’s identity. Article 25 (1) of the UPP, Article 20 of the UŚOZ, and Article 4 of the USIM all require identity verification and acceptance of permission for the provision of health services, in line with the GDPR requirements for data protection (Articles 15–22 of the GDPR, in particular the right to access and correct data errors).

Sharing personal data with unauthorised persons is not allowed under the personal data protection regulations [27]. For this reason, verification is generally performed in person at the medical facility. Patients are required to present one of the following documents: national ID card, passport, driver’s licence, or school/student ID. This approach to identity verification is secure, provided that confidentiality is maintained during the verification process (e.g., no surveillance cameras pointed directly at the bearer, sufficient distance between the registration window and other people in line, etc.). To comply with the law, verification is limited to the presentation of the ID, which means that—legally—these documents cannot be photocopied.

### 3.4. Authentication Issues: User Names and Passwords

In Poland, many patients complain that the registration system requires them to remember “strong” passwords, which are—necessarily—long and complex. However, complex passwords provide only the illusion of data security; moreover, they must be updated every 30 days or so, requiring users to introduce additional criteria (e.g., special signs, digits, or upper case letters), which imposes a further burden. In addition, since many patients make only minor modifications to their password, this reduces data security, as these changes are often easy to predict. Most patients are not able to remember entirely new, strong passwords every month. For this reason, the recommendations made by the CERT (Computer Emergency Response Team) in Poland have been modified. Patients are now required to create longer passwords (about 12 symbols, previously 8) made of full sentences (at least five words), but they do not need to change these passwords every month. Additionally, patients are instructed to avoid using simple passwords, such as those found in common password lists. In many cases, the individual department requires a unique password, which is a major cause of patient frustration. Nonetheless, it is worth noting that the problem of too many complex passwords has a simple solution: password management software or applications. In the case of the Czech Republic, the obligatory use of the “eGovernment” authorisation system has eliminated this issue. Similar efforts are underway in Poland through a security solution that integrates the patient’s national ID (PESEL) to be used with the local e-registration system and the National Node. This system is similar to that used in other countries, such as login.gov in the United States and the eGovernment system in the Czech Republic. This new log-in technology allows patients to register electronically using different electronic identification tools provided both by public and commercial entities, such as trusted profiles, an e-ID card, or other mechanisms widely used in the banking system.

In Poland and the Czech Republic, it is no longer necessary to confirm the patient’s identity in person at the facility, as this is established during the electronic set-up of the account via e-mail and password. Once the account has been created, the patient confirms his/her identity by logging onto the online public service. In this case, no additional passwords or log-in IDs are needed, because a single universal login with a secure password is used. Greater use of the national identification system will allow for an easier, faster, and more secure e-registration process. Moreover, this will eliminate the need for in-person registration, a potentially important advantage for immunocompromised patients. Clearly, the implementation of a modern electronic identification system at the national level will not only improve access to e-services in the health care system in Poland, but also access to private medical facilities, while simultaneously improving data security. Importantly, only identification systems that meet specific requirements are connected to the node, thus ensuring a high level of security, which is further strengthened by the fact that only a single supplier (the national node) is used to confirm identity. This approach to identity verification during e-registration will speed up and facilitate the delivery of health care services.

### 3.5. Text Messages (SMS) Sent by Telephone or Other Modalities

SMS messages, which allow for up to approximately 160 characters, can be used to send reminders through an SMS gateway to patients about upcoming consultations. SMS messages are not secure, and thus should not include any health-related information, and should never reveal the identity of the recipient. The main function of these messages is to remind patients about a scheduled appointment, or to send a code for an e-prescription or an e-referral. This communication channel ensures that the information is sent directly to the patient’s telephone, which is an advantage in terms of speed (the messages reach the recipient in a matter of seconds or minutes), which is often important in a health care context. In addition, studies show that a high percentage of SMS text messages are opened and presumably read. SMS can also be integrated into the IT system, which allows for automated messaging, thereby reducing staff workload. Even though text messages contain no health-related data, since these messages are sent to patients (and include a specific name and surname), the telecommunications operator must, in accordance with Polish Telecommunication Law, implement certain technical measures to ensure the security and integrity of the network. These measures should provide a level of security commensurate with the risk, which means using the appropriate technology while also taking into account the costs of implementing those measures (article 175). These security measures form part of an agreement with the telecommunications operator in accordance with the GDPR regulations. When patients register with the hospital and provide their contact telephone number, they must also verify the telephone number to prevent any mistakes.

SMS is a highly effective and efficient communication tool [28]. However, it is crucial to ensure accurate contact data to make sure that the intended target (the patient) receives the text message. If the registered telephone number is incorrect (or the patient changes the number without notifying the facility), the message could be sent to the wrong person. Both patients and the medical facility must be aware of the risks associated with this type of communication. Clearly, given the low security level of text messages, these should contain only the minimum information necessary and never any personal data, nor any clues that could allow a third party to determine the patient’s identity.

### 3.6. Identification of Inpatients: How to Display the Patient’s Name

Another form of communication in the hospital is how physicians and nurses obtain information about patients in the inpatient ward. Until recently, it was common practice to place a card or document containing the patient’s personal and health-related data at the foot of the bed for easy access and identification. During the rounds, the physician would check these paper documents to see what tests had been performed and to see the prescribed medications. However, the GDPR prohibits the open display of such information. As a result, in part due to accreditation requirements, most medical facilities have started to phase out this practice. Instead, mobile IT equipment (e.g., tablets integrated into the hospital IT system) are commonly used. These tablets can scan barcodes on the patient’s wristband to verify identity and access the medical records.

According to Articles 36 (3) and 36 (5) of the Act of 15 April 2011 on medical activity, all hospitalised patients in Poland must be provided with an identification band, which includes the patient’s name, surname, and date of birth (all of which are coded). The use of a barcode prevents unauthorised persons from identifying the patient. However, this system has an important drawback: a barcode reader is required to access the data. Thus, in an emergency situation, the medical staff may not be able to quickly identify the patient unless they have ready access to a scanner. In both Poland and the Czech Republic, the wristband barcode can only be read at fixed computer stations because wireless scanners are not currently available. When weighing the costs and benefits of barcoded versus open-data wristbands [29], despite the importance of data confidentiality, the most important factor is patient safety, which provides support for recent measures designed to introduce changes to the current law in Poland (Section 63 (3) (a) of the Polish Act on Healthcare Quality). These legislative changes specifically stipulate that the patient’s name, surname, and date of birth must be openly visible on the identification band.

The risk of mistaken identification cannot outweigh data protection [18] and must not interfere with the provision of health care services. To reduce the risk of mistaken identity, the Polish Working Group for the Protection of Personal Data declared that all medical products (including drips) and other treatments administered to the patient should be marked with the patient’s name and surname. Alternatively, these data can be printed on the wristband in such a way that it is difficult for unauthorised persons to read (e.g., location or format of the text, font size). The patient can also cover the wristband to prevent others from reading it. This particular situation reveals the need to find a balance between data protection and the patient’s wellbeing. However, given the numerous benefits of the barcode, prohibiting their use could slow the computerisation process at some facilities and the automatisation process at some health care providers. The barcode enables automatic access to medical records in the IT system without having to manually enter the patient’s full name or identification number. The barcode also makes it possible to electronically order tests, prescribe drugs, and record their use at the patient’s bedside, as well as to check diagnostic test results, thus saving time. Mobile access is essential to obtain real-time patient data, thus obviating the need for stationary computers. That said, the wireless infrastructure requires strong security measures to secure the transmission of sensitive data. In particular, network security requires encryption and enterprise class protection of data transmission, in which the data traffic from the mobile device is encrypted en route to the servers in order to protect sensitive data. Encryption is essential, regardless of the network security level [30,31].

Notwithstanding the benefits of electronic communication and computerisation, these advantages must be weighed against their costs. It is an extremely expensive process and the operating costs to keep everything running smoothly and securely are high. Importantly, these costs are not usually reimbursed by public health insurance (not even partially), but rather must be borne by the provider.

### 3.7. Mobile Devices

The mobile devices used to access data should be “dumb” devices that do not store any data, which are kept on secure servers. Access to these devices should require strong authentication, such as a smart card and PIN or two-factor authentication. Authorisation levels should be appropriate to the work performed, with sufficient permission to allow access to the data needed to provide care. These mobile devices should not be able to access the public network, except for highly restricted access to a few specific Internet resources. The devices should be protected by antivirus software and secured (only encrypted media allowed by the medical facility) [32]. If these security measures are in place, then the risk of unauthorised data access is minimal.

## 4. Discussion

### 4.1. Effective Exchange of Information

Increasingly, our work and personal lives are moving online, accessed through computers, smartphones, tablets, and other devices. As a result, people increasingly expect to be able to use these modern technologies in their interactions with the health care system. In this context, most health care providers have been undergoing a massive transformation in recent years in an effort to modernise their systems, which is clearly necessary given the increasingly important role that data play in health care [33].

The transition to the digital world continues to be slow and cautious in the field of healthcare, mainly due to concerns about the risks associated with confidential health information “floating” around in cyberspace [34]. Patients undoubtedly support the use of modern technologies to improve communication [35] with their doctors, and they also want the physician to have ready—but secure—access to their data from other health care providers or physicians [31].

Apart from the important goal of using new technologies to facilitate personalised communication with the patient, which allows for the rapid, easy exchange of information, it is essential to guarantee the protection of their personal and health-related data [36]. For this reason, it is crucial to strike a balance between excessive data protection and allowing for reasonable and sufficient access to data, especially given that overly zealous restrictions could impede the diagnostic and treatment processes.

### 4.2. Security of Data Exchange in Oncology Practice

Clearly, all data related to health status come under the privacy rights umbrella as established in the GDPR. Although many advances have been made towards standardising the laws surrounding data protection within the EU, much more work needs to be carried out to achieve greater harmonisation among member countries. Indeed, the present study demonstrates, based on our own experience in the practical implementation of national regulations in oncology hospitals, a clear need to implement certain changes. Data breaches could potentially have major negative consequences for the patient and the institution. For this reason, these highly sensitive data must be handled with the utmost care and confidentiality, especially in the current era, in which patients routinely communicate with physicians and the health care facility online through mobile devices.

### 4.3. Benefits and Drawbacks of IT Technologies in Oncology Practice

IT tools make the health care system more efficient, improving the quality and effectiveness of the services provided, which in turn helps patients feel safe. They equip hospitals with tools for remote communication between the patient and the hospital, thus enabling remote registration and patient identification, as well as text messaging with details about upcoming appointments, and websites containing valuable information.

The health care facility’s website has become one the main sources of information for patients. For this reason, it is essential that the website contain all relevant information needed by the patient. The content of hospital websites should be presented in a logical manner, with intuitive navigation to make it easy to obtain information. The website should also allow for electronic registration.

Patients often find authentication to be excessively complicated. For many patients and staff, it is difficult to remember long passwords. However, this difficulty can be overcome by using electronic identification tools provided by public and/or commercial entities, such as trusted profiles, an e-ID card, or other similar mechanisms that are widely used in the banking system.

Inpatient identification is often performed through wrist bands and on labels placed on medical products (e.g., medicines) prepared specifically for a given patient. However, excessive focus on this aspect of patient privacy carries important risks, mainly the risk of mistaken identification due to the use of barcodes to protect the patient’s privacy. In our opinion, the patient’s name and surname should be openly displayed on all medical products (including drips and other treatments). Similarly, inpatients should be clearly identified in the same manner. Although some might argue that this is a privacy breach, we believe that the patient’s safety is the top priority, which is why we advocate for clearly displaying the patient’s name.

SMS is a highly effective and efficient communication tool, provided that it is delivered to the intended patient. Given the low security level of text messages, these should contain only the minimum information necessary, thereby preventing third parties from determining the patient’s identity through the data contained in the message.

Mobile devices should require strong authentication and should not store any data (which should be kept only on secure servers). The use of mobile devices to access public networks should be restricted to a few specialised internet resources.

### 4.4. Study Limitations

The main limitation of this study is that we did not perform a comprehensive review of the literature, but rather focused on our professional, real-life experience at two comprehensive cancer care centres in Europe. As a result, the findings may not be generalisable to other hospitals in other settings. Nevertheless, this study provides concrete examples of the real-world challenges of ensuring data protection and patient privacy in two major hospitals located in the EU.

## 5. Conclusions

In the EU, the processing of patients’ personal data is primarily regulated by the GDPR. We believe that the gradual harmonisation among EU member countries in terms of data protection practices, particularly those that share borders where cross-border mobility is common, could facilitate oncology practice and access to care for patients.

In Poland, personal data protection is regulated by provisions in national law applicable to all medical facilities; by contrast, in the Czech Republic, the treatment of health-related data relies mainly on special GDPR-based standards.

It is essential that the health care facility’s website contain all relevant information needed by the patient. There is also a need to improve electronic communication (for registration, scheduling appointments, etc.) between the patient and the hospital. In the present study, we found that electronic registration was, in many cases, not possible in either of the two countries, mainly because this process depends on the individual hospital, without the support of state authorities and without appropriate legal regulations.

Patient authentication through the use of long passwords that need to be changed frequently has generated much criticism among patients. However, authentication can be simplified by implementing newer technologies that provide the required level of security.

Although the best approach to identifying inpatients remains controversial, we believe that the patient’s name and surname should be openly displayed on all medical products and on the wristband, rather than through a barcode that requires a reader to decipher. This is important to reduce the risks of misidentification, which could have serious consequences.

Text messages sent through SMS should only be used to confirm the time or location of the consultation due to the low level of data security for this communication modality. While mobile devices undoubtedly facilitate medical care and administration, these devices must require strong authentication and should not store any data. In this regard, legal regulations or guidelines for EU member states on IT security are urgently needed to help hospitals ensure the security of patient data. Such regulations would help to standardise medical systems and enable hospitals to work together on improving both patient safety and data security.

## Data Availability

Not applicable.
